# Bilateral metachronous ovarian metastases from clear cell renal carcinoma: a case report

**DOI:** 10.1186/1757-1626-2-7083

**Published:** 2009-06-05

**Authors:** Mauro Albrizio, Alfredo La Fianza, Maria Sole Prevedoni Gorone

**Affiliations:** Department of Radiology, Policlinico San Matteo - University of PaviaPavia (27100)Italy

## Abstract

**Introduction:**

Bilateral ovarian metastases from a clear cell renal carcinoma are uncommon findings and need to be differentiated from primary cancers. Diagnostic imaging and histopathological features are often inconclusive, unless they are combined.

**Case presentation:**

A 56-year-old woman with a history of right radical nephrectomy for a renal clear cell carcinoma diagnosed 10 years earlier was referred for abdominal distension and pelvic pain. Color-Doppler US and Computer Tomography scan revealed the presence of bilateral ovarian masses with regular margins, a low resistance index and poor contrast enhancement. Immunohistochemistry showed positive epithelial membrane antigen, cytokeratin, vimentin and CD10, suggesting clear cells from the previously diagnosed kidney cancer.

**Conclusion:**

Although bilateral metachronous ovarian metastases from clear cell renal carcinoma are a very uncommon finding, they can be considered in the differential diagnosis and investigated with imaging and immunohistochemistry. The 6 cases reported in the literature indicate a good prognosis for this condition.

## Introduction

Ovarian metastases from a renal clear cell carcinoma are uncommon events, which has been described in 19 case reports [1-6]. Some of these secondary lesions are metachronous to the primary tumor, and the vast majority is monolateral [6].

We report a case of bilateral metachronous ovarian metastases from a primary renal clear cell carcinoma (stage I) removed 10 years earlier. Such an unusual finding has been reported in 5 more cases [1-5].

We will discuss clinical presentation, differential diagnosis, imaging features, treatment and prognosis of this condition.

## Case presentation

A 56-year-old Caucasian Italian woman was admitted to our hospital for abdominal distension and pelvic pain. Ten years earlier she had undergone right radical nephrectomy for a stage-I clear cell carcinoma. Following the operation, the patient had not received any adjuvant chemotherapy, but no metastatic cancer was detected at any step of the follow-up.

Clinical examination revealed no obvious abdominal or pelvic masses, while blood tests showed a significant increase in Ca-125 (1006 U/ml).

**Figure 1:**
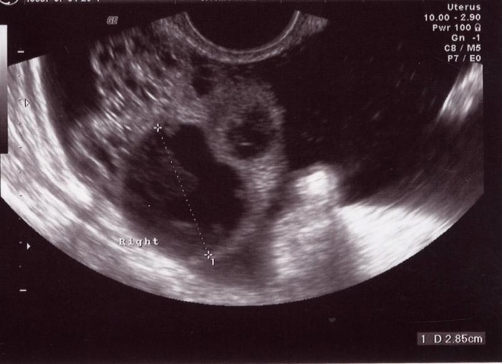
Endocavitary US shows a cystic mass in the right ovary, which appears enlarged. Intraperitoneal free fluid.

A transvaginal ultrasound examination showed ascites in all pelvic quadrants. Both ovaries were small, presenting cystic areas not larger than 3 centimeters (Figure 1). Color-Doppler US showed some vessels with a low resistance index (RI = 0.8).

An abdomino-pelvic multislice CT scan (Sensation 16, Siemens) was performed with acquisitions in the portal phase (70 seconds delay) after I.V. administration of non-ionic iodinate contrast agent (Xenetix 350, Guerbet).

**Figure 2:**
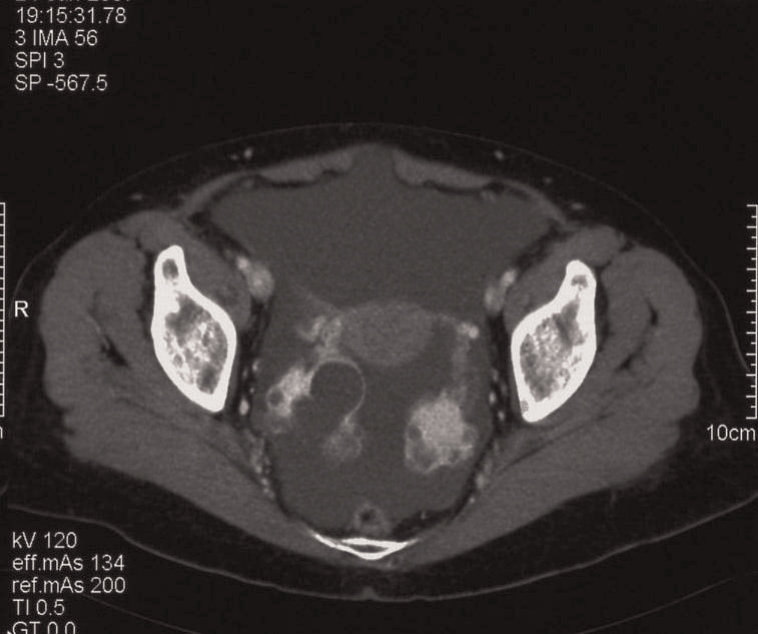
Contrast enhanced CT, portal phase. Presence of bilateral mostly solid parenchymatous ovarian masses with poor contrast enhancement and regular and smooth margins. The scan shows no uterine abnormalities.

The CT study clarified the extent of the ascites (involving the whole abdomen and pelvis) and revealed the presence of ovarian bilateral masses with regular margins and poor contrast enhancement (Figure 2).

There were no tumor recurrences around the nephrectomy site, nor any intra-abdominal or pelvic lymphadenopathies.

A laparoscopic hysterectomy with double oophorectomy was performed. Macroscopically, it looked like both ovaries had been completely replaced by a solid/cystic yellowish mass with multiple hemorrhagic areas.

**Figure 3:**
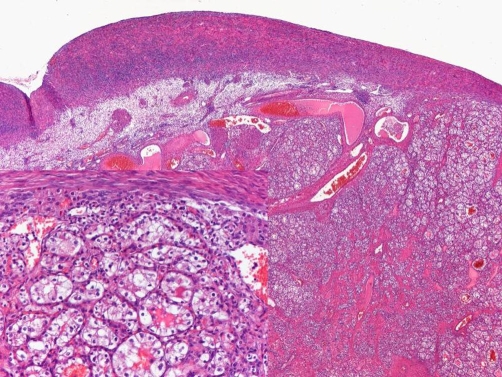
The ovarian parenchyma is almost completely replaced by a clear cell carcinoma consisting in alveolar nests, tubular structures and solid sheaths; there are numerous blood vessels. Tubular structures are composed of polygonal cells with abundant clear cytoplasm and relatively uniform nuclei.

**Figure 4:**
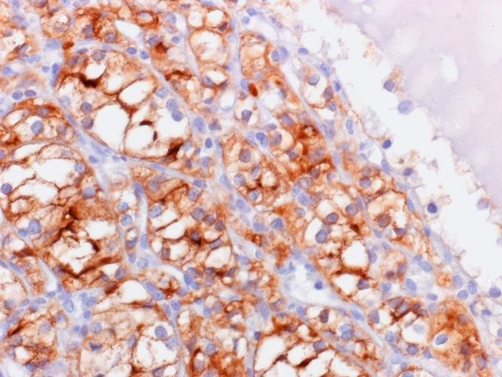
Tumor cells strongly expressing CD10.

On microscopic examination, both masses were populated by polygonal cells with clear cytoplasm and relatively uniform nuclei, some of them exhibiting prominent nucleoli. Tumor cells were arranged in solid sheets and tubular areas, often dilated and containing eosinophilic fluid or blood. In both tubular and solid areas prominent thin-walled blood vessels were recognized (Figure 3). The ascitic fluid was free of malignant cells. Immunohistochemistry of the masses revealed strong and diffuse expression of EMA, cytokeratin, vimentin and CD 10 in tumor clear cells (Figure 4). Immunostainings for cytokeratin 7, cytokeratin 20, inhibin, CA 125, PLAP, estrogen and progesterone receptors, calretinin, WT1, hepatocyte paraffin 1 and CD 34 were uniformly negative for tumor cells.

Based on morphologic findings and cancer cell phenotype, the diagnosis was bilateral ovarian metastases from the known renal clear cell carcinoma.

Chemotherapy as an adjuvant treatment was offered, but the patient declined in view of unpleasant collateral effects.

She is currently alive with no evidence of disease 19 months after the diagnosis of relapse.

## Discussion

In the literature there are 19 cases of renal clear cell carcinoma metastasized to the ovaries, 5 of which are bilateral and metachronous [1-5].

Table 1 summarizes demographic features of the other bilateral metastases. Presenting symptoms were aspecific in all cases. All patients were still alive after an average follow-up of 27 months (ranging from 6 to 48 months).

**Table 1:** Summary table of the literature cases of metachronous bilateral ovarian metastases from clear cell renal carcinoma

Author	Age	Time of recurrence (yrs)	Presentation	Follow up/ Survival (months)	Treatment (in chronological order)	Outcome
Vara	66	14	Right abdominal pain	24	- R-Nephrectomy	NED
					- Total Hysterectomy + double adnexectomy	
Spencer	42	*	Bilateral flank pain	48	- Total Hysterectomy + double adnexectomy	AD
					- L-Nephrectomy	
					- Radiation therapy to metastases (other organs)	
Adachi	46	3	Vaginal bleeding	36	- L-Nephrectomy	NED
					- Total Hysterectomy + double adnexectomy	
Valappil	61	7	Dysuria	24	- L-nephrectomy	AD
					- Splenectomy, omentectomy for recurrence (after 5 years)	
					- Double adnexectomy	
					- Interferon	
Vorder-Bruegge	64	11	Bowel obstruction/ Ascitis	6	- R-Nephrectomy	NED
					- Double adnexetomy	
Our case	56	10	Pelvic pain	15	- R-Nephrectomy	NED
					- Total Hysterectomy + double adnexectomy	

NED (no evidence of disease); AD (alive with disease).

* Clear cell renal carcinoma detected 6 months after bilateral salpingo-oophorectomy.

Two patients were found to have a disease relapse [2,4]. Spencer [2] reported metastases to the parotid gland, thyroid and brain at 18 months, which were treated with surgery and radiation therapy; the patient was alive with disease 48 months later. The patient presented by Valappil [4] developed tumor recurrences to pelvic and para-aortic nodes 8 months after ovarian metastases and was treated with interferon but metastases persisted.

Drug treatment after the finding of adnexal metastases was administered only in two of the literature reports [3,4]; an inhibitor of cell proliferation (interferon alpha) was used in both cases. Survival does not significantly change in treated versus untreated patients (35 versus 26 months). Based on these outcomes the prognosis of the metastatic cancer in question could be considered favorable.

One of the uncommon features in our case is the finding of ascites without peritoneal and parenchymal spread, as well as lymph node sparing at the operation site despite the presence of metastases.

The differential diagnosis of ovarian malignancies in these cases can be awkward, particularly when we need to differentiate primary from secondary lesions. We could not detect any unquestionable morphological sign of ovarian metastases even using different imaging techniques [4,8].

Several attempts have been made to try to find some typical features associated with the radiological appearance of ovarian metastases.

Some authors indicate that ovarian metastases are mostly solid, small and bilateral [7].

In a separate study performed on a cohort of patients with stomach and colonic cancers, we observed that bilateral ovarian masses with clear-cut margins, associated with peritoneal carcinomatosis and with no ascites, are of metastatic nature in most cases [8]. Some of the morphologic features in this case are in agreement with those findings (bilateral presentation, clear-cut margins), unlike the presence of ascites and the absence of peritoneal carcinomatosis.

However, the radiological characteristics that could make a safe difference between ovarian metastases and clear cell carcinoma of the kidney have not yet been established: not enough experimental studies have been performed so far and literature reports are too few to allow unquestionable conclusions to be drawn.

For the time being, with the help of pathologic findings (structural features and immunohistochemical profile, i.e. positivity for CD10, cytokeratin and CA 125) we can make the safer differential diagnosis.

We would like to reiterate the importance of a past medical history positive for renal tumor, which was the first fundamental element of suspicion in our case.

In conclusion, the unreliable nature of metastatic renal cancer makes it worth considering this possibility in the differential diagnosis of any suspicious abdominal mass.

## List of abbreviations

CA: Cancer Antigen
CT: Computer Tomography
EMA: Epithelial membrane antigen
IV: Intravenous
PLAP: Placental Alkaline Phosphatase
RI: Resistance index
